# Development and Genomic Evaluation of a Novel Functional Fermented Milk Formulated with *Lactobacillus delbrueckii* Strains and Jujuba Kernel Powder for Potential Neuroprotective Effects

**DOI:** 10.3390/foods14244264

**Published:** 2025-12-11

**Authors:** Amel A. Ibrahim, Nancy M. El Halfawy, Yuqi Zhang, Ya Liu, Xirui Zhang, Shuxin Zhou, Jianquan Kan, Muying Du

**Affiliations:** 1 College of Food Science, Southwest University, Chongqing 400715, China; amal.ibrahim@alexu.edu.eg (A.A.I.); zyq20021226@email.swu.edu.cn (Y.Z.); m19562217600@163.com (Y.L.); siri_zhang997@163.com (X.Z.); zsx18200391607@email.swu.edu.cn (S.Z.); 2 Department of Dairy Science and Technology, Faculty of Agriculture, Alexandria University, Alexandria 21545, Egypt; 3 Department of Botany and Microbiology, Faculty of Science, Alexandria University, Alexandria 21511, Egypt; nancy.elhalfawy@alexu.edu.eg; 4 Chinese-Hungarian Cooperative Research Centre for Food Science, Southwest University, Chongqing 400715, China; 5 Chongqing Key Laboratory of Specialty Food Co-Built by Sichuan and Chongqing, Chongqing 400715, China

**Keywords:** functional fermented milk, neurodegenerative diseases, probiotic, acetylcholine inhibitor, jujube kernel powder

## Abstract

This study aimed to isolate and screen lactic acid bacteria (LAB) with neuroprotective potential for food applications. Fifteen strains were screened for probiotic potential properties, γ-aminobutyric acid (GABA) production, and acetylcholinesterase (AChE) inhibitory activity. *Lactobacillus delbrueckii* AY8 and AY15 demonstrated the strongest probiotic potential, AChE inhibitory activity, and GABA production. Whole-genome sequencing confirmed genes linked to these probiotic and neuroprotective traits. To assess their functionality in a food matrix, the strains were used as adjunct cultures in fermented milk with and without jujube kernel powder (JP). Fermentation with the AY8 strain in JP-fortified milk significantly increased bioactive compounds, resulting in higher total phenolic content (235.75 mg GAE/g), flavonoids (114.07 mg RE/g), and superior antioxidant activity (110.24 mg Ascorbic equivalent/100 g). This biotransformation led to a remarkable increase in AChE inhibition, with the AY8-fermented sample achieving 30.66% inhibition, significantly higher than the JP control (18.27%) and the plain control (12.30%). The combination also improved the product’s viscosity and sensory profile. This study highlights the successful discovery of novel *L. delbrueckii* strains, whose application in a food model, when combined with a plant-based supplement, creates a functional food with enhanced neuroprotective potential, underscoring the role of microbial metabolism in food functionality.

## 1. Introduction

The global rise in neurodegenerative diseases, particularly dementia, constitutes a significant socioeconomic and healthcare challenge [1], a situation exacerbated by ageing populations and the absence of curative therapies [2]. As the seventh leading cause of death worldwide, dementia currently affects over 57 million individuals, with 10 million new cases annually and projections surpassing 152 million by 2050 [3]. The pathogenesis of conditions like Alzheimer’s and Parkinson’s disease is fundamentally multifactorial, driven by oxidative stress [4], neuroinflammation [5], enzymatic dysregulation such as acetylcholinesterase (AChE) activity [6], and gut–brain axis disruption [7].

In response, innovative strategies for leveraging natural products are urgently needed. Probiotics have emerged as promising therapeutic agents against neurodegenerative diseases [8]. Specific strains, including *Lactobacillus delbrueckii*, demonstrate potent antioxidant, antimicrobial, and anti-inflammatory properties [9]. Their neuroprotective potential is underscored by their capacity to mitigate oxidative stress, synthesize γ-aminobutyric acid (GABA), a critical inhibitory neurotransmitter for cognitive stability [10], and inhibit AChE, thereby preserving acetylcholine levels essential for memory and learning [11]. Furthermore, exopolysaccharides from lactic acid bacteria strains like *L. delbrueckii* exhibit anti-inflammatory and immunomodulatory effects, protecting neurons by modulating MAPK/NF-κB signaling pathways [12], with the gut–brain axis serving as a critical conduit for these benefits [13].

Concurrently, plant-based bioactives offer a complementary neuroprotective approach. In China, jujube (*Ziziphus Jujuba* Mill.) kernel, a major agricultural by-product with an annual production exceeding three million tons [14], represents an underutilized resource. Traditionally considered inedible [15], jujube kernels are rich in bioactive compounds like flavonoids, alkaloids, and polysaccharides [16]. These compounds are responsible for their documented antioxidant, anti-inflammatory, and antimicrobial properties, which contribute to significant neuroprotective and anti-insomnia effects in vivo, as confirmed for constituents such as 6′″-feruloylspinosin [16].

Therefore, integrating jujube kernel powder (JP), noted for its potent bioactive profile, with specifically selected lactic acid bacteria (LAB) strains possessing inherent neuroprotective properties, presents a promising and innovative strategy for functional food development. This study aims to select traditional LAB isolates based on their acetylcholinesterase inhibitory activity, GABA production capacity, and probiotic properties for use as adjunct cultures in a novel functional fermented milk. JP is utilized as a prebiotic supplement to enhance the product’s functional properties, and the novel formulation was evaluated for its potential neuroprotective effects through an in vitro study.

## 2. Materials and Methods

### 2.1. Isolation of LAB and Screening for Acetylcholinesterase Inhibition Potential

Twenty-five samples of traditional fermented dairy products were collected and stored at 4 °C (Table S1). For isolation, samples were cultured in skimmed milk at 30, 37, and 42 °C until coagulation. The samples were then streaked onto M17 for *Lactococci* (Baidei, Lhoka, China) and MRS (Aobox biotechnology, Beijing, China) for *Lactobacilli* agar plates, then incubated anaerobically using the Gas pack system (Anaeropack-MicroAero, Niigata, Japan) at the same temperatures. Purified isolates were phenotypically evaluated and stored at −80 °C for further investigation [17].

Sterile cell-free filtrate (CFF) obtained from 48 h cultures was prepared by centrifugation (10,000× *g*, 10 min, 4 °C), followed by neutralization and filtration through a sterile 0.22 µm membrane filter. CFFs were tested for acetylcholinesterase inhibitory (AChEI) activity using a microplate assay [18]. Donepezil hydrochloride (DRS; Shanghai Aladdin Biochemical Technology, Shanghai, China) and galantamine hydrobromide (GRS; Macklin, Shanghai, China) were used as positive controls. All experiments were performed in triplicate and repeated three times. The percentage of AChE inhibition was calculated using the equation:

**AChEI**
**(%) = [(Blank OD − Sample OD)/Blank OD] × 100**

whereas Blank is phosphate-buffered saline (PBS; 50 mM) for calculating the AChEI% of standards, Blank is culture media (MRS or M17) for calculating the AChEI% of CFF.

The experiments were performed in triplicate (*n* = 3), and the results are presented as the mean percentage of inhibition ± standard deviation.

### 2.2. Probiotic Potential and Safety Characteristics

The fifteen LAB isolates, which revealed AChEI potential, were evaluated for their functional probiotic potential.

#### 2.2.1. Assessment of Acid, Pepsin, and Pancreatin Tolerance

The tolerance to gastric conditions and intestinal conditions was evaluated according to Madian et al. [19]. Briefly, overnight bacterial cultures were harvested by centrifugation (5000× *g*, 10 min, 4 °C), washed twice in PBS, and resuspended in simulated gastric juice to a final concentration of ~10^9^ CFU/mL. The gastric juice consisted of PBS at pH 3.0, supplemented with pepsin (Shanghai Aladdin, Shanghai, China) at a concentration of 1:10,000. In the gastric phase, the suspension was incubated at 37 °C for 0 and 3 h. Following the intestinal phase, 1 mL of the culture was transferred to 9 mL of simulated intestinal juice (pH 8.0, containing 1 mg/mL pancreatin) and incubated at 37 °C for 0 and 4 h. Optimum culture media inoculated with each strain separately under optimum conditions were used as a biotic control. Sterile simulated gastric juice without bacterial inoculation was used as an abiotic control. The survival rate (SR) under stress was calculated for each isolate relative to its own specific control as:

**Survival Rate (%) = (Final log_10_ CFU mL^−1^ (under stress)/Initial log_10_ CFU mL^−1^ (its biotic control) × 100**

The isolates’ acid resistance was categorized into four groups [20] according to their survival rate: Susceptible (<10%), Moderate resistance (10–60%), Good resistance (60–80%), and very good resistance (>80%).

#### 2.2.2. Bile Salt Tolerance

Bile tolerance was evaluated according to Guo et al. [21]. Overnight cultures were inoculated into MRS broth with and without 0.3% (*w*/*v*) bile salts. Uninoculated MRS broth with and without 0.3% bile salts served as abiotic controls and were used to blank the spectrophotometer. These cultures were incubated at 37 °C, and their growth was recorded by measuring absorbance at OD_600_ hourly up to 24 h using a spectrophotometer (Jinghua, China), with uninoculated media used as a blank. Based on their survival rate in the presence of bile salts, the isolates were categorized into four groups according to Chateau et al. [22].

#### 2.2.3. Antibacterial Activity Against Gastrointestinal Pathogens

The in vitro antimicrobial activity was assessed using the agar well diffusion method against *Salmonella enterica* serovar *Typhimurium* ATCC33110 and *Escherichia coli* NCTC12900. Pathogens (10^9^ CFU/mL) were inoculated separately into specific culture media, and 6 mm-diameter wells were created. One hundred microliters of sterile neutralized CFS (nCFS) were added to each well and then incubated for 24 h at 37 °C. Ampicillin (10 mg/mL) was used as a positive control, while uninoculated MRS broth was used as a negative control. The average diameter of the inhibition zone was measured in millimeters (mm).

#### 2.2.4. Detection of γ-Aminobutyric Acid (GABA) Production Gene

Total DNA was extracted from an exponential-phase bacterial culture using the TIANamp Bacteria DNA Kit (Tiangen Biotech, Beijing, China). The presence of the *gad* gene was assessed by polymerase chain reaction (PCR) with specific primers (gad-F/gad-R, Sangon Biotechnology, Shanghai, China) using a Direct PLABCR kit (Sangon Biotechnology, Shanghai, China). The reaction was performed in a Bio-Rad thermal cycler, and the resulting amplicons were analyzed via 1% (*w*/*v*) agarose gel electrophoresis. Controls included *L. plantarum* SWU-ZFT6 (positive) and *L. rhamnosus* DSM 20021 (negative). The PCR protocol followed the conditions described by Langa et al. [23].

#### 2.2.5. Hemolytic Activity

The hemolytic activity of the LAB isolates was assessed to evaluate their potential safety for applications [24]. Each isolate was streaked onto blood agar plates supplemented with 7% (*v*/*v*) defibrinated sheep blood. The plates were then incubated under microaerophilic conditions at 37 °C for 48 h. Following incubation, the plates were examined for hemolysis.

#### 2.2.6. Antibiotic Susceptibility Testing (AST)

The antibiotic susceptibility of the LAB strains was determined by the disk diffusion method, using ten types of antibiotic discs: Ampicillin (10 µg), Ceftriaxone (30 µg), Tetracycline (30 µg), Gentamicin (10 µg), Penicillin (10 U), Erythromycin (15 µg), Ciprofloxacin (5 µg), Chloramphenicol (30 µg), Lincomycin (2 µg), and Sulfamethoxazole (25 µg). The plates were incubated at optimal conditions for 24 h. The diameter of the inhibition zones was measured in mm, and data were classified as susceptible (**S**), intermediate (**I**), or resistant (**R**) according to CLSI guidelines [25].

### 2.3. Molecular Identification Using 16S rRNA

Molecular identification of LAB isolates was performed through 16S rRNA gene sequencing. Purified genomic DNA was used as a template for amplification of the 16S rRNA gene using the specific primers 27F (5′-AGAGTTTGATCCTGGCTCAG-3′) and 1492R (5′-CGGTTACCTTGTTACGACTT-3′). The amplified amplicons were sequenced. The obtained sequences were then analyzed for similarity using the Basic Local Alignment Search Tool (BLAST) available through the National Center for Biotechnology Information (NCBI) database.

### 2.4. Genotypic Characterization of AY8 and AY15 Strains

#### 2.4.1. Extraction of Genomic DNA

Bacterial strains AY8 and AY15 were inoculated into MRS broth and cultured at 37 °C for approximately 12 h at 150 rpm. The cell biomass was harvested by centrifugation (10 min, 12,000× *g*), and genomic DNA was extracted using the Bacterial/Fungal DNA extraction kit (Magnetic Beads, Changzhou, China) according to the manufacturer’s protocol.

#### 2.4.2. Whole Genome Sequencing

Genome sequencing was outsourced using a combination of PacBio and Illumina sequencing platforms. For Illumina sequencing, genomic DNA was used to construct the sequencing library for each strain. Illumina sequencing libraries were prepared from the sheared fragments using the Nextera XT Library Prep Kit (Illumina, San Diego, CA, USA). The prepared libraries were then used for paired-end Illumina sequencing (2 × 150 bp) on the Illumina NovaSeq 6000 platform (Illumina, San Diego, CA, USA). For PacBio sequencing, genomic DNA was fragmented, purified, end-repaired, and ligated with SMRT bell sequencing adapters (Pacific Biosciences, Menlo Park, CA, USA). Next, the PacBio library was prepared and sequenced on one SMRT cell using standard methods.

#### 2.4.3. Bioinformatics Analysis

The data generated from PacBio and Illumina platforms were used for bioinformatics analysis. The analyses were performed using the online platform of Majorbio Cloud Platform from Shanghai Majorbio Bio-pharm Technology. The short reads and HiFi reads were assembled to construct complete genomes using Unicycle (V0.4.8) [26] and using Pilon (V1.22). The coding sequences of the chromosome and plasmid were predicted using Glimmer or Prodigal (V2.6.3) [27] and GeneMarkS [28], respectively.

### 2.5. Using Selected Strains as Adjunct Culture in the Functional Fermented Milk Process

#### 2.5.1. Preparation of Cultures

The adjunct strains were activated through three successive subcultures for AY8 and AY15. The inoculated milk was then incubated at 42 °C for 18 h to achieve a final concentration of approximately 2 × 10^8^ CFU/mL. This target concentration was selected as it exceeds the minimum probiotic viability threshold (10^6^–10^7^ CFU/mL) recommended for functional foods and provides a sufficient bacterial load for metabolite production [29].

The base material for yoghurt production was prepared by reconstituting whole milk powder (Yili, China) in water. According to the manufacturer, the powder contained 25% protein, 28% fat, and 42% carbohydrates (on a dry-matter basis). The final composition of the reconstituted milk base was analytically determined using AOAC [30] and was as follows: 12.0% total solids, 2.76% protein, 3.11% fat, and 4.6% carbohydrate. This standardized base was used for all subsequent yoghurt preparations. Reconstituted milk was then divided into two main batches. One batch was supplemented with 2% (*w*/*v*) jujube seed powder (JP), obtained from Nanjing Tongrentang Health Industry, China, with a composition of 97.5% total solids, 1.12% protein, and 4.06% fat. The second batch, without JP, served as the control. Both batches were subsequently heat-treated at 90 °C for 5 min [31]. After heat treatment, the milk was cooled to the fermentation temperature of 42 °C. Each main batch (with and without JP) was further subdivided into four treatments, resulting in a total of eight experimental groups. **C1**: Inoculated directly with a commercial yogurt starter culture (*Lactobacillus* Yogurt starter, Beijing Chuanxiu Technology, China) and incubated at 42 °C until coagulation at pH 4.70 ± 0.1 was achieved, **T1**: Pre-fermented with strain AY8 (1 h, 42 °C) before the addition of the commercial starter culture, **T2**: Pre-fermented with strain AY15 (1 h, 42 °C) before the addition of the commercial starter culture, **T3**: Pre-fermented with a combination of strains AY8 and AY15 (1 h, 42 °C) before the addition of the commercial starter culture. Samples **C2**, **T4**, **T5**, and **T6** are processed under the same conditions, except JP. Fermentation was terminated when a target pH of 4.70 ± 0.1 was reached. The resulting fermented milk was then gently stirred, sugar 5%, cooled, and stored at 4.0 ± 0.5 °C for 14 days for subsequent analysis.

#### 2.5.2. Physicochemical Analysis

The physicochemical properties of the functional fermented milk were analyzed throughout the cold storage period. The pH was measured (PHS-3E, Shanghai Instrument & Electronics Scientific Instruments Co., Ltd., Shanghai, China). Titratable acidity (expressed as % lactic acid), total solids %, and total nitrogen content were analyzed by the Kjeldahl method according to AOAC [30]. Fat content was determined using a filter bag technique (ANKOM XT15 Extractor, ANKOM Technology, Macedon, NY, USA).

#### 2.5.3. Viscosity and Sensorial Evaluation of Functional Fermented Milk

The apparent viscosity of the samples was determined according to the principles of ISO 7884-2 [32] during cold storage. Records were performed using a rotational viscometer (NDJ-8S, Fangrui Instrument, Shanghai, China) equipped with spindle 3 at a speed of 60 rpm at 10 °C. Sensory attributes, i.e., appearance, texture, aroma, taste, and overall acceptability of functional fermented milk samples were evaluated by well-trained ten panelists consisting of 6 males and 4 females from the College of Food Science, Southwest University, China. Samples were prepared 24 h post-fermentation and stored at 4 °C until evaluation. Approximately 30 mL of each sample was served at room temperature (20 ± 2 °C) in identical, colorless cups labelled with random codes. Each panelist evaluates all samples in a randomized order, and water was provided for palate cleansing between samples. A 9-point hedonic scale was used where 1 represented “dislike extremely,” 5 represented “neither like nor dislike,” and 9 represented “like extremely” [33].

#### 2.5.4. Survival of LAB

Lactic acid bacteria were enumerated in MRS at 37 °C for 72 h under anaerobic conditions during cold storage [34].

#### 2.5.5. Bioactive Compounds and Antioxidant Activity

Twenty grams of the functional fermented milk sample was accurately weighed. The sample was mixed with n-hexane in a 1:3 (*w*/*v*) ratio [35]. Then, the mixture was subjected to sonication in an ultrasonic water bath (15 min, 30 °C), maintaining a constant temperature. The defatted residue was subsequently extracted for polyphenols and other antioxidant compounds using a methanol-water solution. A mixture of 80% aqueous methanol (*v*/*v*) was added to the sample in a 1:10 (*w*/*v*) ratio [36], with some modifications. The extraction was performed with continuous agitation for 24 h at 4 °C. After the extraction period, the mixture was first centrifuged (10,000× *g*, 15 min, 4 °C) to pellet insoluble particulates. The supernatant was then filtered to obtain a clear crude extract.

Total Phenolic Content (TPC) was analyzed using a modified Folin–Ciocalteu method [37] with some modifications. Briefly, 300 µL of extract was reacted with diluted Folin–Ciocalteu reagent and sodium carbonate solution (7.5%). After a 30 min incubation, absorbance was measured at 765 nm. TPC was calculated from a gallic acid standard curve (0.02–2.0 mg/mL) and expressed as mg GAE/g.

The Total Flavonoid Content (TFC) was determined using the aluminium chloride colorimetric method [38]. The assay was performed by sequentially adding 150 µL of 5% NaNO_2_ and, after 6 min, 150 µL of 10% AlCl_3_ to a mixture of 500 µL of extract and 2 mL of distilled water. Six minutes after the addition of AlCl_3_, 2 mL of 4% NaOH was added. The volume was made up to 5 mL with distilled water. After a 15 min incubation period for chromogen development, the absorbance was read at 510 nm. A standard curve was generated using rutin (250–1000 µg/mL), and the TFC was calculated and expressed as milligrams of rutin equivalents per 100 g of sample (mg RE/100 g).

##### Antioxidant Activity

The extract was dissolved in 10 mL of 80% methanol. This concentration was used for the subsequent determination of antioxidant activity via the DPPH assay [39]. The DPPH radical scavenging activity was determined according to the following procedure: 300 µL of the methanolic extract was combined with 2.7 mL of a 0.2 mM DPPH methanolic solution. After thorough vortexing, the mixture was incubated for 30 min at room temperature in the dark. The absorbance was then measured at 517 nm using a UV-Vis spectrophotometer (Thermo Electron Corporation, Giheung, Republic of Korea), with a methanol blank as a reference. The scavenging activity of the samples was quantified based on the ascorbic acid standard curve(0–15 µg/mL) and is expressed as mg of Ascorbic Acid Equivalents per 100 g of sample.

The ferric reducing antioxidant power was determined according to [39]. Briefly, 1 mL of each diluted extract was mixed with 2.5 mL of phosphate buffer (0.1 M, pH 6.6) and 2.5 mL of potassium ferricyanide solution (1% *w*/*v*). The mixture was incubated at 50 °C for 20 min. Subsequently, 2.5 mL of trichloroacetic acid (10% *w*/*v*) was added. Finally, 2.5 mL of the resulting mixture was combined with 2.5 mL of distilled water and 0.5 mL of ferric chloride solution (0.1% *w*/*v*). After standing for 30 min, the absorbance was measured at 700 nm. All assays were performed in triplicate. The reducing power was expressed as a percentage, calculated using a standard formula.

##### Acetylcholinesterase (AChE) Inhibition

The extract was dissolved in 2 mL of 50 mM phosphate buffer containing 0.1% dimethyl sulfoxide (Shanghai Aladdin, Shanghai, China). The phosphate buffer provides the optimal pH for compounds without inhibiting the enzyme [40,41].

### 2.6. Statistical Analysis

Data were expressed as the mean of three replicates ± standard deviation, except for the sensorial evaluation test, where ten replicates were used. A one-way analysis of variance (ANOVA). Where the ANOVA revealed significant differences (*p* < 0.05), Duncan’s new multiple range test was applied to compare the means between sample groups. All statistical analyses were performed using the IBM SPSS Statistics package 24.0 (SPSS, Chicago, IL, USA). The figures were generated using GraphPad Prism (V9.5.0).

## 3. Results and Discussion

### 3.1. AChE Inhibition by LAB Strains

**Figure 1:**
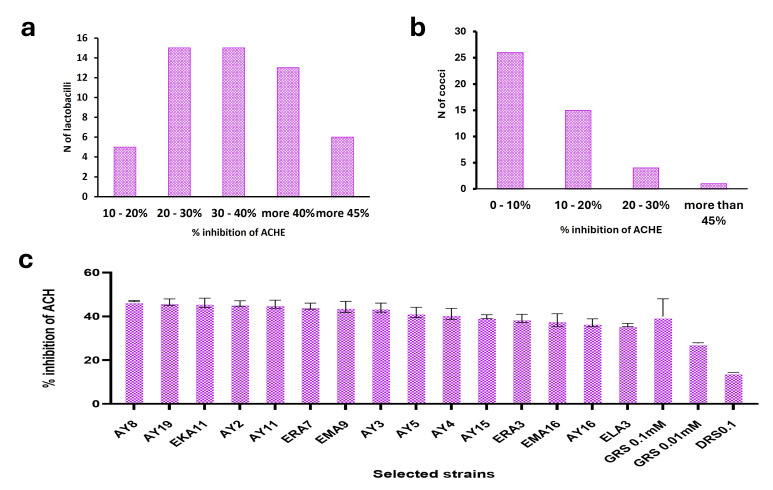
Theinhibition ability of lactic acid bacteria isolates against acetylcholinesterase. (**a**) Inhibition ability of lactobacilli (**b**) Inhibition ability of cocci (**c**) %ACH inhibition of lactic acid bacteria and enterococcus strain. **GRS**: Galantamine reference standard; **DRS**: Donepezil reference standard.

Of the ninety-five LAB isolates obtained from traditional dairy products, forty-eight (50.5%) were classified as lactobacilli and forty-seven (49.5%) as cocci (Tables S2 and S3). When screened for AChE inhibitory activity, fourteen lactobacilli isolates demonstrated high inhibition (Figure 1a; Table S2). Among the cocci, only one enterococcus isolate, EKA11, revealed high inhibition (Figure 1b; Table S3), while the rest exhibited minimal to moderate activity. Figure 1a,b show the distribution of % inhibition from a single representative experiment across all isolates. The complete dataset with mean values and standard deviations from triplicate experiments (*n* = 3) for each isolate is provided in Supplementary Tables S2 and S3. Isolate AY8 revealed the highest potent AChE inhibition (46.99% ± 0.26), followed by lactobacilli isolate AY19 (46.50% ± 1.46) and *Enterococcus* isolate EKA11 (46.19% ± 2.11) (Table 1). The inhibitory activity of these isolates surpassed that of the standard drug donepezil (0.1 mM), which showed 14.4% ± 0.05 inhibition (Figure 1c). The activity was also higher than that of galantamine (39.96% ± 8.07) at 0.1 mM and significantly exceeded the inhibition reported for *Levilactobacillus brevis* P109 (9.85% ± 0.14%) in a previous study by Qadah et al. [18]. Previous studies indicate that increasing the level of the cholinergic neurotransmitter has a positive effect on cognitive functions and alleviates the symptoms of Alzheimer’s disease [42]. Given that conventional pharmacological inhibitors of this enzyme are associated with hepatotoxicity and potential lethality [43], the development of natural inhibitors presents an innovative therapeutic and prophylactic strategy.

### 3.2. Probiotic Potential Properties

For LAB to function effectively as probiotics, 15 LAB isolates evaluated for probiotic potential properties revealed significant variation in their tolerance. After incubation at pH 2.0, isolates AY15 and AY16 demonstrated exceptional acid resistance. Statistical analysis confirmed that the differences in survival rates under these conditions, which ranged from 47.66% to 99.62%, were significant (*p* < 0.05). Furthermore, the isolates exhibited a high degree of tolerance to intestinal stressors. When exposed to a trypsin-containing environment, survival rates were notably high, ranging from 46.66% to 100%. Survival rates for all strains were calculated relative to strain-specific biotic controls incubated under optimal conditions, which accounted for the inherent growth variations between different isolates. Additionally, in the presence of 0.3% bile salts, five isolates maintained high survival rates of more than 60%, while isolates AY8 and EMA9 showed moderate resistance (Table 1). Based on their superior and consistent performance across all critical tests, exceptional acid tolerance, high survival in trypsin, and robust bile salt resistance, both isolates AY15 and AY16 were identified as the most promising candidates. Growing evidence underscores the significant role of probiotics in modulating neurological health and cognitive function, primarily through the gut–brain axis [44,45].

Probiotics provide neurological benefits through several key mechanisms. They reduce neuroinflammation by suppressing pro-inflammatory cytokines, fortify the blood–brain barrier, and help balance neurotransmitter levels. A critical function is their ability to combat oxidative stress, a major factor in neurodegenerative diseases. Evidence from preclinical studies shows that probiotics can improve cognitive performance and reduce the accumulation of amyloid-beta plaques in the brains of Alzheimer’s disease model mice [46]. These benefits extend to humans, as clinical trials have demonstrated that probiotic supplementation can lead to significant cognitive improvements in Alzheimer’s patients and help slow neurocognitive decline [47].

### 3.3. Antibacterial Activity

**Figure 2:**
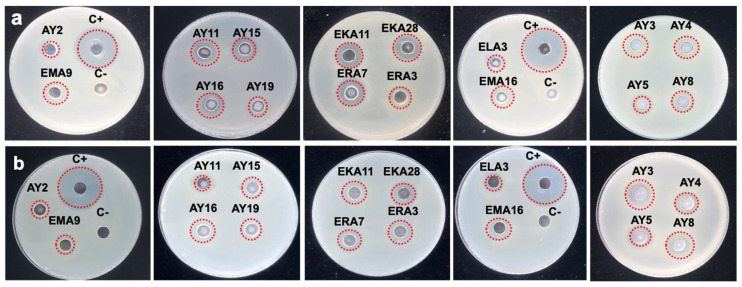
Antimicrobial activity of selected isolates against (**a**) *Escherichia coli* (10^9^ CFU/mL) and (**b**) *Salmonella enterica* serovar *Typhimurium* (10^9^ CFU/mL). **C+**: Ampicillin (10 mg/mL), **C-**: culture media.

The inhibitory activity of the lactic acid bacterial isolates against *E. coli* and *Salmonella enterica* serovar *Typhimurium* is highly relevant in the context of the gut–brain axis, as these pathogens are implicated in mechanisms that can contribute to neuroinflammation and neurodegeneration [48]. Neutralized CFS revealed significant antimicrobial activity against strains under investigation (Table 1; Figure 2). The diameters of inhibition zones against *E. coli* ranged from 11.25 ± 0.95 to 18.66 ± 0.57 mm. Moreover, the diameters ranged from 11.33 ± 0.57 to 19.33 ± 1.52 against *Salmonella enterica* serovar [49]. Certain strains of *E. coli* are known to produce amyloid-curli proteins, and *Salmonella* infections can trigger a cascade of systemic inflammation [50]. Both are linked to the misfolding and aggregation of neuronal proteins such as α-synuclein and amyloid-beta, which are hallmarks of Parkinson’s and Alzheimer’s disease [51]. Therefore, by effectively suppressing the proliferation of *E. coli* and *Salmonella enterica* serovar *Typhimurium*, the lactic acid bacterial strain contributes to the maintenance of gut homeostasis. This action helps prevent pathogen-induced gut dysbiosis, reduces the load of pro-inflammatory molecules, and subsequently mitigates a key driver of brain dysfunction, which may contribute to neuroprotective effects mediated through the gut–brain axis [52].

### 3.4. Detection of the Glutamate Decarboxylase (GAD) Gene

**Figure 3:**
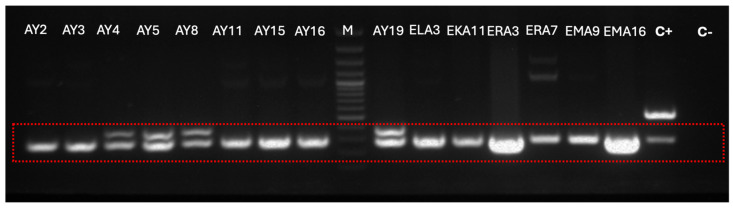
Representative gel electrophoresis for the detection of gad gene in all tested strains. M: DNA marker (100–3000 bp), **C+**: L. plantarum SWU-ZFT6 (GDMCC 66290), **C-**: *L. rhamnosus* DSM 2002.1.

The PCR confirmation of the *gad* gene in all selected lactic acid bacteria and enterococci strains confirms their inherent capacity for GABA production (Figure 3). This finding, coupled with their established AChE inhibitory activity, suggests a synergistic dual mechanism for neuroprotection. While AChE inhibition enhances cholinergic signaling to support cognitive function, the concomitant GABA production addresses the critical issue of excitotoxicity in neurological disorders [53].

It is important to contextualize the reported disruptive role of endogenous GABAergic systems in Alzheimer’s disease progression [54]. This impairment likely reflects a loss of GABAergic inhibitory tone rather than a detrimental effect of GABA itself. The bacterially derived GABA may thus serve to restore this compromised inhibition, counteracting glutamate-mediated excitotoxicity and helping rebalance the excitatory-inhibitory equilibrium essential for neuronal integrity [55]. This multi-target approach represents a promising strategy that moves beyond symptomatic relief by addressing core pathological processes in neurodegenerative diseases.

### 3.5. Hemolytic Activity and AST

All selected LAB isolates under investigation exhibited a γ-hemolytic phenotype, confirming their non-hemolytic behavior. Furthermore, AST of lactic acid bacteria isolates against tested antibiotics demonstrated variable resistance and sensitivity patterns (Table 2). Briefly, a high level of susceptibility was observed toward ampicillin, ceftriaxone, erythromycin, chloramphenicol, and ciprofloxacin. In contrast, resistance was observed against gentamicin and penicillin in several strains, while tetracycline exhibited a strain-dependent response ranging from susceptibility to intermediate resistance. Notably, resistance to lincomycin and sulfamethoxazole was less frequent, although certain isolates displayed intermediate resistance (Table S4). Oh, and Jung [56] reported that the absence of antibiotic resistance and hemolytic activity is the basic requirement for selecting a new safe probiotic isolate.

### 3.6. Molecular Identification

The 16S rRNA genes of the lactic acid bacterial isolates were amplified and sequenced for strain-level identification (Table 2). Among the isolates, all isolated from yak milk were assigned to *Lactobacillus delbrueckii*, strains with different subspecies identification.

### 3.7. Genomic Features of AY8 and AY15

**Figure 4:**
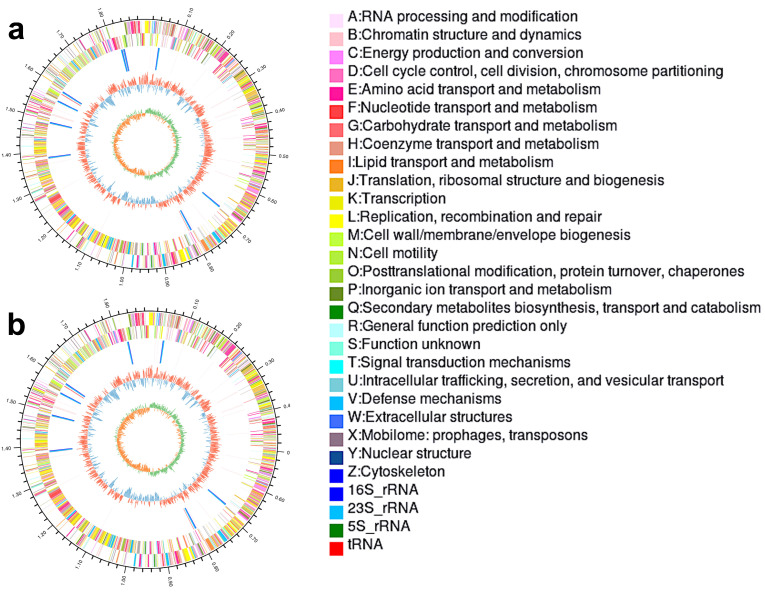
Circular genomic map of (**a**) *Lactobacillus delbrueckii* subsp. *bulgaricus* AY8 (**b**) *Lactobacillus delbrueckii* subsp. *allosunkii* AY15.

AY8 and AY15 were selected for whole genome sequencing and in-depth genomic investigation due to their superior performance across probiotic tolerance assays and the highest AChE inhibition potential. The genomes revealed circular chromosomes with a total length of 1,897,414 bp and 1,890,519 bp, respectively. The average GC content of both strains was about 49.60%. The chromosome of AY8 contains 1913 CDS, 27 rRNA, and 95 tRNA. Moreover, AY15 contains 1907 CDS, 24 rRNA, and 88 tRNA (Figure 4). Pathogenicity analysis revealed the absence of clinically relevant antibiotic resistance genes, critical virulence factors, and toxin-related genes, making them a viable option for use as a probiotic supplement [57].

#### 3.7.1. Determination of Probiotic-Associated Genes

Based on the genome annotation of *L. delbrueckii* subsp. *bulgaricus* AY8 and *L. delbrueckii* subsp. *allosunkii* AY15, several probiotic-associated genes were identified (Table 3). The genomes revealed the presence of adhesion and biofilm-associated genes, including LPxTG-motif protein, sortase A (*srtA*), and enolase (*eno*), which are essential for specific interaction between bacterial surface components and the host epithelial cell surface receptors [58]. Moreover, genes conferring bile and acid tolerance (*cfa*, *nagB*, *ppa*, *nhaC*, *napA*) were consistently detected in both strains, supporting their persistence within the GIT, allowing them to arrive in viable amounts sufficient to promote their interactions and beneficial effects with the specific-host sites of action [59]. Stress adaptation mechanisms were also evident, with a full complement of heat shock proteins (*htpX*, *hrcA*, *grpE*, *dnaK*, *dnaJ*, *groES*, *groEL*), which play a role at high temperatures and revealed a defense mechanism against sudden heat shock stress. Moreover, the cold stress regulator (*cspA*) is present in both genomes, which helps to survive in temperatures lower than the optimum growth temperature [60]. In addition, antimicrobial activity was confirmed by the detection of the class III RamC lanthionine bacteriocin, a heat-stable bacteriocin that catalyzes the hydrolysis of bacterial cell walls, causing cell lysis and death [61].

#### 3.7.2. Determination of AChE Inhibitory Genes

Several genes encoded in the genome sequences of AY8 and AY15 can be directly or indirectly linked to the inhibition of AChE, which may protect against neurodegenerative diseases. The AY8 and AY15 strains revealed genes involved in the synthesis of neuroactive molecules, particularly the *gadC* gene, which encodes glutamate-GABA. This gene is involved in GABA export and glutamate import [62]. The byproduct of the decarboxylation reaction of glutamic acid acts as an inhibitory neurotransmitter in the human central nervous system [63]. Moreover, pyroglutamyl peptidase, encoded by *pcp*, is a highly specific membrane-bound thyrotropin-releasing hormone-degrading enzyme [64]. It acts as a tripeptide with multiple homeostatic functions in the brain, directly influencing neuronal excitability and potentially mood balance [65]. Furthermore, the presence of riboflavin synthesis-associated genes (*ribE*, *ribD*) indicated the potential of AY8 to synthesize riboflavin (vitamin B2) and provide an explanation for its higher potential to inhibit AChE in vitro in comparison with AY15 (which lacks these genes). Indeed, riboflavin is essential for myelin synthesis and contributes to neuroprotection by supporting cellular energy balance and reducing oxidative stress [66]. Moreover, riboflavin supports antioxidant defense (e.g., glutathione reductase, catalase) and mitochondrial energy production, both compromised in Alzheimer’s disease [67]. This genomic distinction provides a strong mechanistic lead for future studies aimed at experimentally validating the role of riboflavin production in modulating neuroactive properties of probiotic strains.

In addition, both strains harbor *folC*, encoding dihydrofolate synthase, which is essential for the synthesis of folate (vitamin B9). A previous study reported that sufficient folate intake may reduce the risk of Alzheimer’s disease occurrence [68]. The presence of thioredoxin (Trx) in both isolates highlights an additional neuroprotective trait, as it plays a role in maintaining cellular redox homeostasis. Trx has been recognized as a promising therapeutic agent for the prevention and treatment of a wide range of neurodegenerative, neuroinflammatory, and neuro-oxidative stress-related disorders [69]. Moreover, both strains harbor a wide array of exopolysaccharide cluster genes. Several studies have explored the potential protective effects of lactic acid bacteria against Alzheimer’s disease; however, a recent study specifically attributed these effects to the exopolysaccharides produced by LAB through mechanisms such as free radical scavenging, modulation of peroxidation products, and apoptosis [1].

The final selection of *Lactobacillus delbrueckii* AY8 and AY15 for the fermentation trials was based on a comprehensive evaluation of their complementary functional attributes, as detailed in Table 1. AY8 (*L. delbrueckii* subsp. *bulgaricus*) was selected as the primary neuroprotective strain due to its superior AChE inhibitory activity (46.99 ± 0.26%) and GABA production potential, representing the highest observed bioactivity among all screened isolates. While demonstrating moderate bile salt tolerance (49.47%), its exceptional neuroprotective profile justified its inclusion. The selection of AY15 (*L. delbrueckii* subsp. *lactis*) over the closely competing strain AY16 was strategically based on its balanced combination of neuroprotective potency and probiotic robustness. Although both strains exhibited excellent stress tolerance, AY15 demonstrated significantly higher AChE inhibition (39.86% vs. 37.08%), substantial antimicrobial activity against *Salmonella enterica* serovar *Typhimurium* (15.0 mm inhibition zone vs. 0 mm for AY16), and superior gastric acid survival (99.94% vs. 97.29% at pH 2.0). Furthermore, genomic analysis revealed EPS gene clusters in AY15, predicting enhanced technological properties for product texture development. This strategic pairing ensures optimal synergy between AY8’s targeted neuroprotective efficacy and AY15’s multifunctional probiotic robustness, creating an ideal foundation for developing advanced functional fermented milk with demonstrated health benefits.

### 3.8. Effect of Selected Strains and JP on Fermented Milk

#### 3.8.1. Physicochemical Properties

**Figure 5:**
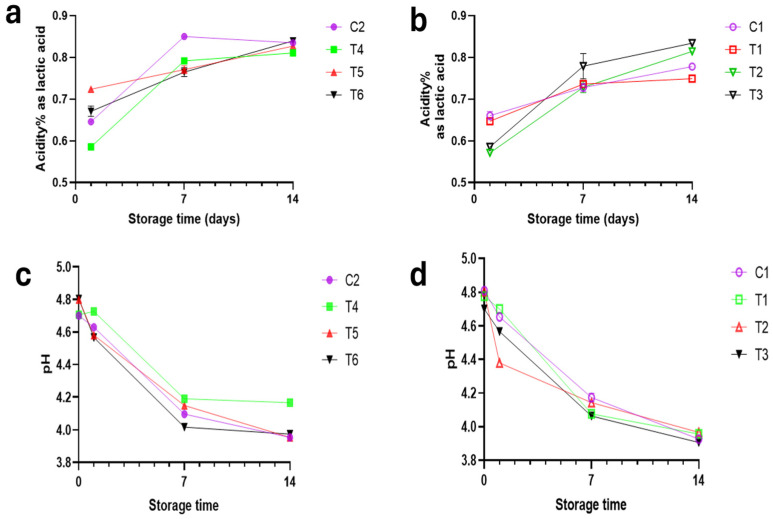
Acidity percentage (%) and pH of functional fermented milk during cold storage (**a**) acidity % of functional fermented milk without JP (**b**) acidity of fermented milk with 2% JP (**c**) pH of functional fermented milk without JP (**d**) pH of fermented milk with 2% JP. **C2**: Control fermented milk by using commercial starter culture; **T4**: fermented milk by using AY8 as adjunct culture; **T5**: fermented milk by using AY15 as adjunct culture; **T6**: fermented milk by using mix of strains AY8 & AY15 as adjunct culture; **C1**: Control fermented milk by using commercial starter culture supplemented 2% JP; **T1**: fermented milk by using AY8 as adjunct culture supplemented 2% JP; **T2**: fermented milk by using AY15 as adjunct culture supplemented 2% JP; **T3**: fermented milk by using mix of strains AY8 & AY15 as adjunct culture supplemented 2% JP.

The addition of 2% jujube kernel powder (JP) did not significantly alter the pH compared to the control (*p* > 0.05), a finding attributed to the buffering capacity of the JP’s protein content [70], which moderates the typical pH drop during fermentation [71]. Despite this, a significant increase in acidity was observed across all samples during storage (*p* < 0.05), indicating sustained metabolic activity of the lactic acid bacteria. This acid production was more pronounced in JP-fortified samples, suggesting that the powder’s prebiotic components supported enhanced bacterial activity and acidification (Figure 5). Nutritional analysis confirmed that JP fortification led to a marginal increase in fat content (Table 4), consistent with the use of defatted JP (residual fat: 5.1%). This approach is advantageous as it minimizes potential issues with wax ester digestibility while concentrating on the desired bioactive compounds. Furthermore, a significant increase in the protein content of the fermented milk was confirmed upon JP supplementation.

#### 3.8.2. Viscosity and Sensorial Evaluation

**Figure 6:**
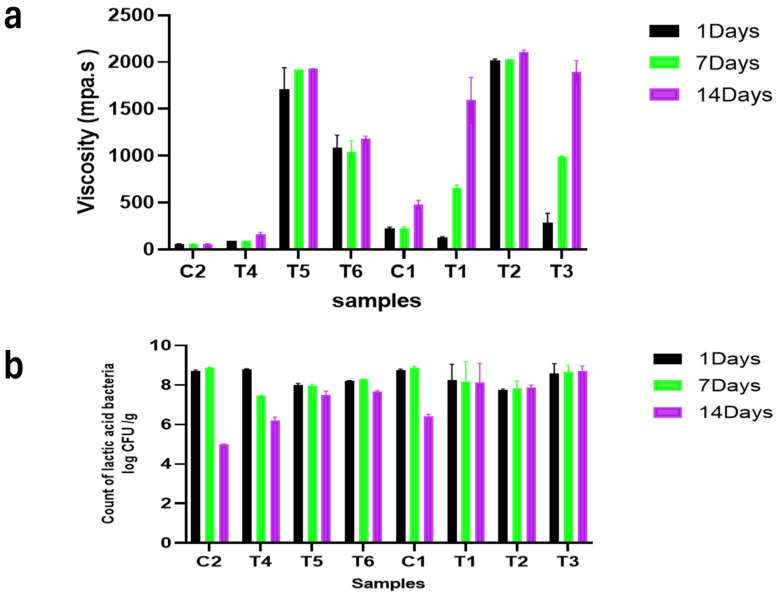
(**a**) Viscosity and (**b**) survival of lactic acid bacteria of fermented milk during cold storage. **C2**: Control fermented milk by using commercial starter culture; **T4**: fermented milk by using AY8 as adjunct culture; **T5**: fermented milk by using AY15 as adjunct culture; **T6**: fermented milk by using mix of strains AY8 & AY15 as adjunct culture; **C1**: Control fermented milk by using commercial starter culture supplemented 2% JP; **T1**: fermented milk by using AY8 as adjunct culture supplemented 2% JP; **T2**: fermented milk by using AY15 as adjunct culture supplemented 2% JP; **T3**: fermented milk by using mix of strains AY8 & AY15 as adjunct culture supplemented 2% JP.

Significant differences in viscosity were observed among the treatments (*p* < 0.05). As shown in Figure 6a, the sample fermented with the exopolysaccharides-producing strain AY15 and fortified with JP exhibited the highest viscosity. Whole-genome sequencing confirmed that the AY15 genome harbors key gene clusters for exopolysaccharides biosynthesis (Table 3), directly linking this genetic potential to its functionality as a natural biothickener [72]. JP fortification independently increased viscosity, attributable to its high protein content, predominantly glutelin and albumin, which contribute to gel network formation [73]. The maximum viscosity observed in sample T2 suggests a powerful synergy between the jujube kernel powder (JP) and the AY15 strain. Given that whole genome sequencing of AY15 revealed a complete set of genes for exopolysaccharide (EPS) biosynthesis and considering that the fibrous components of JP could potentially serve as prebiotic substrates, we hypothesize that an enhancement in EPS production may be a contributing factor to the improved rheological properties. However, direct quantitative measurement of EPS is required to conclusively confirm this mechanism. This represents a limitation of the current study and a key objective for our immediate future work.

These rheological improvements directly enhanced sensory perception. The mean sensory scores for all functional fermented milk treatments are presented in Table 4. The results indicate that the choice of bacterial strain and the addition of jujube powder (JP) significantly influenced the product’s sensory profile. Regarding texture, treatments incorporating the AY15 strain received significantly higher scores (*p* < 0.05), with a mean value of 8.15 ± 0.63, compared to 6.2 ± 1.03 for the control. This underscores the positive impact of AY15’s in situ exopolysaccharide production on mouthfeel, likely contributing to a thicker and smoother perception.

JP supplementation further improved texture scores for both strains, confirming its role as a functional texture modifier. In contrast, analysis of Aroma scores showed no significant differences (*p* > 0.05) between any of the treatments, including those with JP. All mean scores fell within a narrow, acceptable range (8.65–8.9). This indicates that neither the adjunct cultures nor the JP introduced any detectable off-flavors, which is critical for consumer acceptance. This finding aligns with Zhang et al. [74], who reported that JP can enhance the aroma profile of fermented foods without detrimental effects.

A positive and significant trend was observed for taste. The treatments containing both AY8 and JP (T1) and AY15 and JP (T2) achieved the highest taste score of 8.2 ± 0.75 and 8.2 ± 0.48, respectively, which was significantly higher (*p* < 0.05) than the control C2 (7.3 ± 0.95). This suggests that JP contributed desirable sensory notes, such as characteristics, slight sweetness, and complexity, which improved overall taste acceptance. This is consistent with the benefits of plant-based fortification seen in other dairy products [75].

Furthermore, Treatments T1 (AY8 + JP) and T2 also demonstrated high overall acceptability (8.4 ± 0.52 and 8.1 ± 0.74, respectively), forming a top statistical group with T3 from which they were not significantly different (*p* > 0.05). This indicates that multiple formulations, specifically those combining either the AY8 or AY15 strain with JP, were highly successful and preferred by the panellists over the controls. The results underscore that the addition of JP was a key factor in enhancing the sensory profile, and its synergy with both candidate bacterial strains yielded products with superior overall acceptability.

### 3.9. Effect of JP as a Prebiotic

It was observed from Figure 6b that the number of lactic acid bacteria decreased during storage. However, the counts remained above 10^6^ CFU/mL in all samples except the control (C2), which is essential for probiotic efficacy [76]. The most significant finding of this study is the stark contrast in lactic acid bacteria (LAB) viability between the control and jujube seed powder (JP)-fortified yogurts. The control sample, formulated with a low-protein milk base, suffered a catastrophic four-log reduction in LAB counts, highlighting the severe stress of post-acidification in a nutrient-limited environment. In direct contrast, all JP-supplemented samples demonstrated markedly enhanced bacterial survival. This compelling evidence indicates that JP provides a critical protective effect, likely through prebiotic nutrient supply and antioxidant activity, establishing its role not merely as a fortification but as a functional stabilizer that significantly improves the probiotic potential of yogurt. The reduction in lactic acid bacteria counts was not significant (*p* > 0.05) in samples supplemented with JP, indicating a pronounced protective effect on bacterial viability. The viability of lactic acid bacteria counts in the presence of JP can be directly attributed to its unique biochemical composition. Jujube kernels are a rich source of polysaccharides and various bioactive compounds, including flavonoids and saponins [77,78]. These components likely function through two primary mechanisms. The first is the Prebiotic Effect due to its content of polysaccharides, which are known to act as prebiotics, serving as a fermentable substrate for lactic acid bacteria [79]. The second is antioxidant protection, referring to the content of bioactive compounds (e.g., flavonoids) that possess potent antioxidant properties [80]. During storage, lactic acid bacteria are subjected to oxidative stress, which can damage cell membranes and DNA, leading to cell death. The antioxidants in jujube powder are posited to mitigate this stress, thereby preserving cell membrane integrity and enhancing the survival rate of the bacteria. The maintenance of lactic acid bacteria counts above 10^6^ CFU/mL is crucial, as it not only ensures the biochemical stability of the fermented product but also preserves its potential probiotic benefits for gut health. Therefore, the incorporation of jujube kernel powder presents a viable natural strategy for enhancing the shelf-life and functional quality of probiotic-containing products.

### 3.10. Evaluation of Fermented Milk for Potential Neuroprotective Effects

The results presented in Table 5 clearly demonstrate significant differences (*p* ˂ 0.05) in total phenol and flavonoid content among the tested samples. The functional jujube fermented milk sample T1 showed the highest levels of key bioactive compounds and functional activity. It recorded the greatest concentration of total phenols (235.75 mg GAE/100 g) and total flavonoids (114.07 mg RE/100 g), which contributed to its superior antioxidant capacity, as evidenced by the highest DPPH scavenging activity (110.24 mg Ascorbic equivalent/100 g), and FRAP assay (99.07%). Furthermore, a significant increase in bioactivity was also observed in the samples (T4 and T5) fermented with the selected strains (AY8 and AY15), even in the absence of jujube powder, compared to the control (C2). The enhancement can be directly attributed to the metabolic activities of the employed strains, which are known to bioconvert complex phenolic compounds into simpler, more bioavailable flavonoids or to synthesize additional flavonoid molecules during the fermentation process [81]. The results clearly revealed that each strain (AY8 and AY15) was more effective when used individually than when combined in a co-culture in treatments (T3 and T6).

Most notably, T1 demonstrated the strongest inhibition of AChE at 30.66%, a critical activity for potential neuroprotective effects. The fact that sample C1, which contained jujube powder but was not fermented with a selected strain, showed significantly lower inhibition (18.27%) underscores that the specific metabolic activity of the bacterial strain is the primary driver behind the enhanced bioactivity. This is further supported by the strong AChE inhibition in samples T4 and T5, which achieved 24.49% and 22.45% inhibition, respectively, despite their lower overall phenolic and flavonoid content, confirming that the strains themselves contribute potent inhibitory effects.

While flavonoids are well-documented AChE inhibitors [82], our results suggest that the selected strains do not merely increase the quantity of these compounds. Instead, they likely biotransform the substrate to release or produce specific, highly potent inhibitors, which could include a particular subset of flavonoids, bioactive peptides, GABA, or other microbial metabolites. The superior performance of the T1 formulation indicates a synergistic effect where the jujube matrix provides optimal precursors for these strain-specific bioconversions.

These findings highlight the potential of combining selected fermentative strains with jujube kernel powder to create a synergy that significantly enhances the functional, particularly neuroprotective, properties of food products.

In this study was the superior performance of individual strains, particularly AY8, over the combination of two strains as co-culture in enhancing bioactive compounds and AChE inhibitory activity. This apparent antagonistic effect in the mixed culture provides valuable insights into microbial interactions, which can be explained through several mechanistic perspectives supported by recent scientific literature. The observed reduction in bioactivity may be attributed to resource competition, where both strains, belonging to the same species, potentially compete for identical, limited nutrients in the milk matrix, thereby restricting the metabolic expression of one or both strains [83]. Furthermore, environment-mediated suppression could play a significant role, wherein rapid acidification by one strain may create suboptimal pH conditions that inhibit the growth or enzymatic activity of the other [84]. Additionally, negative physiological interactions might be involved, where metabolic byproducts such as bacteriocins or organic acids from one strain directly inhibit the other [85].

Given that AY8 showed exceptional performance in monoculture, it is plausible that the presence of AY15 in the co-culture negatively regulates the specific metabolic pathways in AY8 responsible for generating neuroprotective compounds. This finding aligns with recent studies emphasizing that simply combining high-performing strains does not necessarily yield synergistic effects and may even lead to functional antagonism [86]. Consequently, our results underscore the critical importance of strain-specific compatibility and a detailed understanding of microbial metabolic interactions when designing effective multi-strain functional foods.

## 4. Conclusions

This study successfully isolated novel probiotic strains with significant in vitro neuroprotective potential, demonstrating AChE inhibition and GABA production. The strains proved suitable as adjunct cultures for fermented milk production. Firstly, it moves beyond conventional probiotic screening by employing a genomics-guided approach to select strains with a genetic predisposition for neuroprotective functions, such as GABA production. Secondly, it demonstrates that JP is not merely a functional ingredient but a synergistic component that enhances both the viability of the probiotics and the sensory acceptability of the final product, addressing a common hurdle in functional food development. Most significantly, we provide in vitro evidence that our formulated fermented milk exhibits acetylcholinesterase inhibitory activity that surpasses that of the standard drug donepezil, positioning it as a promising, natural dietary intervention for cognitive health.

Despite these promising results, several limitations of this study must be acknowledged. The primary limitation is that the neuroprotective effects are based on in vitro assays; the efficacy of the product needs to be validated in vivo animal models and, ultimately, human clinical trials. Furthermore, while genomic analysis predicted the potential for bioactive metabolite production, GABA quantification was not determined at the actual levels in the final product. Finally, the long-term stability of these bioactivities during product storage remains unverified.

In conclusion, this study establishes a solid foundation for the use of tailored probiotic strains and functional plant ingredients as a viable strategy for creating neuroprotective foods. Future work will focus on quantifying the specific bioactive metabolites, elucidating their precise mechanisms of action, and validating the therapeutic potential in vivo.

## Figures and Tables

**Table 1:** Inhibition ACH%, Antibacterial activity, and surviving percentage of LAB strains in 0.3% bile salt, low pH 2.0 and 4.0, and in the artificial gastric juices.

Strain Code	Mean ± SD *							
	Inhibition ACH%	Antimicrobial Activity Inhibition Zone Diameter (mm)		Survival Rate %				
		*E. coli* NCTC12900	*S. Typhimurium* ATCC13311	Bile Salt Tolerance (0.3% Oxgall)	Low pH		Gastric Acid Tolerance	
					pH 2.0	pH 4.0	Pepsin	Trypsin
**AY2**	45.85 ± 1.24 ^a^	16.33 ± 0.57 ^bc^	14.66 ± 0.57 ^ef^	28.80 ± 0.20 ^j^	34.09 ± 2.07 ^f^	94.58 ± 4.57 ^ab^	89.51 ± 0.69 ^b^	99.76 ± 0.25 ^a^
**AY3**	43.99 ± 2.10 ^abc^	18.16 ± 0.28 ^a^	17.75 ± 0.5 ^bc^	12.67 ± 0.13 ^m^	0 ± 0.0 ^h^	89.54 ± 0.52 ^ab^	62.16 ± 0.14 ^e^	91.95 ± 4.7 ^de^
**AY4**	41.12 ± 2.53 ^cd^	16.83 ± 0.28 ^b^	17.16 ± 0.76 ^cd^	8.77 ± 0.30 ^n^	0 ± 0.0 ^h^	97.96 ± 0.73 ^ab^	57.97 ± 1.83 ^f^	96.53 ± 5.04 ^abcd^
**AY5**	41.79 ± 2.36 ^bcd^	15.33 ± 0.57 ^cd^	18.66 ± 0.57 ^ab^	14.75 ± 0.45 ^l^	0 ± 0.0 ^h^	89.72 ± 1.23 ^ab^	55.14 ± 4.7 ^f^	97.37 ± 2.27 ^abc^
**AY8**	46.99 ± 0.26 ^a^	14.66 ± 0.57 ^de^	17.5 ± 0.5 ^cd^	49.47 ± 1.60 ^f^	45.90 ± 0.52 ^d^	89.32 ± 1.80 ^ab^	87.34 ± 2.1 ^b^	99.00 ± 1.07 ^ab^
**AY11**	45.55 ± 1.86 ^ab^	16.66 ± 0.57 ^b^	17.0 ± 0 ^cd^	24.49 ± 0.81 ^k^	23.43 ± 0.40 ^g^	89.94 ± 0.61 ^ab^	89.52 ± 0.51 ^b^	97.33 ± 1.82 ^abc^
**AY15**	39.86 ± 0.88 ^def^	14.0 ± 1.0 ^ef^	15.0 ± 0 ^ef^	90.02 ± 0.48 ^d^	99.68 ± 1.12 ^a^	99.94 ± 0.91 ^ab^	99.62 ± 0.64 ^a^	100 ± 0.0 ^a^
**AY16**	37.08 ± 1.82 ^ef^	11.25 ± 0.95 ^i^	0 ± 0 ^i^	98.74 ± 0.66 ^a^	84.95 ± 0.04 ^b^	97.29 ± 1.04 ^ab^	99.19 ± 0.33 ^a^	99.19 ± 0.33 ^ab^
**AY19**	46.50 ± 1.46 ^a^	13.83 ± 0.76 ^efg^	19.33 ± 1.52 ^a^	36.68 ± 0.33 ^i^	0 ± 0.0 ^h^	100 ± 0.0 ^ab^	65.49 ± 3.6 ^d^	95.97 ± 2.92 ^abcd^
**ELA3**	36.04 ± 1.89 ^f^	15.0 ± 0 ^de^	15.0 ± 0 ^ef^	1.14 ± 0.91 ^o^	0 ± 0.0 ^h^	73.77 ± 1.23 ^b^	47.66 ± 2.03 ^g^	47.66 ± 2.03 ^g^
**EKA11**	46.19 ± 2.11 ^a^	18.66 ± 0.57 ^a^	14.0 ± 0 ^f^	37.8 ± 0.39 ^h^	34.87 ± 0.76 ^f^	99.11 ± 4.39 ^ab^	97.35 ± 2.02 ^a^	87.26 ± 1.14 ^f^
**ERA3**	39.08 ± 1.9 ^def^	13.0 ± 0 ^fg^	16.66 ± 0.57 ^d^	94.49 ± 0.12 ^b^	48.23 ± 0.47 ^c^	99.06 ± 1.71 ^ab^	97.51 ± 0.29 ^a^	94.06 ± 4.41 ^cd^
**ERA7**	44.66 ± 1.42 ^abc^	12.75 ± 0.95 ^gh^	11.33 ± 0.57	88.83 ± 0.24 ^e^	46.08 ± 0.52 ^d^	97.26 ± 0.84 ^ab^	89.27 ± 0.47 ^b^	89.27 ± 0.47 ^ef^
**EMA9**	44.35 ± 1.95 ^abc^	11.66 ± 1.15 ^hi^	0 ± 0 ^h^	44.46 ± 0.19^g^	0 ± 0.0 ^h^	97.75 ± 2.0 ^ab^	72.09 ± 2.09 ^c^	94.65 ± 2.5 ^bcd^
**EMA16**	38.27 ± 3.88 ^def^	14.66 ± 0.577 ^de^	15.66 ± 0.57 ^e^	92.09 ± 0.45 ^c^	41.76 ± 0.08 ^e^	101.27 ± 1.13 ^a^	99.1 ± 1.14 ^a^	99.13 ± 1.67 ^ab^
**GRS0.1 mM**	39.96 ± 8.07 ^de^	-						
**GRS0.01 mM**	27.64 ± 0.32 ^g^	-						
**DRS0.1 mM**	14.40 ± 0.05 ^h^	-						

* Mean of three replicates ± standard deviation. Means that are followed by the same letter in the row and the same capital letter in the column did not differ significantly (*p* ˂ 0.05).

**Table 2:** Identification of lactic acid bacteria isolates by 16S rRNA gene sequencing and their safety properties.

Strain Code	Identification by 16S rRNA	Hemolysis	Number of Antibiotic-Sensitive
**AY2**	*Lactobacillus delbrueckii* subsp. *bulgaricus*	no hemolysis	7/10
**AY3**	*Lactobacillus delbrueckii* subsp. *allosunkii*	no hemolysis	7/10
**AY4**	*Lactobacillus delbrueckii* subsp. *bulgaricus*	no hemolysis	7/10
**AY5**	*Lactobacillus delbrueckii* subsp. *allosunkii*	no hemolysis	7/10
**AY8**	*Lactobacillus delbrueckii* subsp. *bulgaricus*	no hemolysis	8/10
**AY11**	*Lactobacillus delbrueckii* subsp. *allosunkii*	no hemolysis	8/10
**AY15**	*Lactobacillus delbrueckii* subsp. *allosunkii*	no hemolysis	7/10
**AY16**	*Lactobacillus delbrueckii* subsp. *allosunkii*	no hemolysis	6/10
**AY19**	*Lactobacillus delbrueckii* subsp. *bulgaricus*	no hemolysis	8/10
**ELA3**	*Limosilactobacillus fermentum*	no hemolysis	4/10
**EKA11**	*Enterococcus faecium*	no hemolysis	9/10
**ERA3**	*Lactiplantibacillus plantarum*	no hemolysis	6/10
**ERA7**	*Limosilactobacillus fermentum*	no hemolysis	9/10
**EMA9**	*Limosilactobacillus reuteri*	no hemolysis	3/10
**EMA16**	*Lactiplantibacillus plantarum*	no hemolysis	6/10

**Table 3:** Predicted probiotic genes in *Lactobacillus delbrueckii* strains AY8 and AY15 genome sequences based on KEGG annotation.

Function	Genes	Product	AY8	AY15
**Adhesion** **capacity and biofilm formation**				
	LPXTG	Surface protein (LPXTG motif)	+	+
	*srtA*	Sortase A [EC:3.4.22.70]	+	+
	*eno*	Enolase [EC:4.2.1.11]	+	+
**Bile tolerance**				
	*cfa*	Cyclopropane-fatty-acyl-phospholipid synthase [EC:2.1.1.79]	+	+
	*nagB*	Glucosamine-6-phosphate deaminase [EC:3.5.99.6]	+	+
	*ppa*	Manganese-dependent inorganic pyrophosphatase [EC:3.6.1.1]	+	+
**Acid tolerance**				
	*nhaC*	Tyrosine transporter, NhaC family	+	+
	*napA*	Na^+^/H^+^ antiporter NapA	+	+
**Temperature tolerance**				
	*htpX*	Heat shock protein HtpX [EC:3.4.24.-]	+	+
	*hrcA*	Heat-inducible transcriptional repressor	+	+
	GRPE	molecular chaperone GrpE	+	+
	*dnaK*	Chaperone protein DnaK	+	+
	*dnaJ*	Chaperone protein DnaJ	+	+
	*grpE*	Heat shock protein GrpE	+	+
	*groES*	Heat shock protein 10 kDa family chaperone GroES	+	+
	*groEL*	Heat shock protein 60 kDa family chaperone GroEL	+	+
**Cold stress**				
	*cspA*	Cold shock protein	+	+
**Antimicrobial activity**				
	*ramC*	Lanthionine synthetase LanKC (class III) [EC:3.13.2.4]	+	+
**Inhibitory neurotransmitter**				
	*gadC*	Glutamate: GABA antiporter	+	+
	*pcp*	Pyroglutamyl-peptidase [EC:3.4.19.3]	+	+
**Vitamins synthesis**				
	*ribE*	Riboflavin synthase [EC:2.5.1.9]	+	-
	*ribD*	diaminohydroxyphosphoribosylaminopyrimidine deaminase	+	-
	*folC*	Dihydrofolate synthase [EC:6.3.2.12 6.3.2.17]	+	+
**Antioxidants**				
	*trxA*	Thioredoxin	+	+
**Exopolysaccharides biosynthesis protein**				
	*epsA*	Protein tyrosine kinase modulator	+	+
	*epsB*	Protein-tyrosine kinase [EC:2.7.10.3]	+	+
	*epsG*	Transmembrane protein EpsG	+	+
	*epsF*	Glycosyltransferase EpsF [EC:2.4.-.-]	+	+
	*epsJ*	Glycosyltransferase EpsJ [EC:2.4.-.-]	+	+
	*exoY*	Exopolysaccharide production protein ExoY	+	+
	*licD*	Lipopolysaccharide choline phosphotransferase [EC:2.7.8.-]	+	+

Where (+) Present; (-) Absence.

**Table 4:** Chemical analysis and sensorial evaluation of functional fermented milk.

Tests	Samples							
	C2	T4	T5	T6	C1	T1	T2	T3
Mean ± SD *								
**Chemical analysis**								
T.S%	17.36 ± 0.04 ^c^	17.30 ± 0.08 ^c^	17.22 ± 0.13 ^c^	17.39 ± 0.10 ^c^	18.92 ± 0.19 ^b^	19.34 ± 0.07 ^a^	19.33 ± 0.04 ^a^	19.30 ± 0.02 ^a^
Fat%	3.11 ± 0.2 ^ab^	3.05 ± 0.18 ^ab^	3.0 ± 0.15 ^b^	3.08 ± 0.1 ^ab^	3.21 ± 0.02 ^ab^	3.29 ± 0.05 ^a^	3.18 ± 0.09 ^ab^	3.19 ± 0.08 ^ab^
Protein%	2.70 ± 0.01 ^b^	2.69 ± 0.0 ^b^	2.70 ± 0.01 ^b^	2.69 ± 0.0 ^b^	2.85 ± 0.01 ^b^	2.85 ± 0.02 ^b^	2.86 ± 0.0 ^b^	2.94 ± 0.01 ^a^
**Sensorial evaluation**								
Appearance	7.0 ± 1.25 ^a^	7.0 ± 1.05 ^a^	8.0 ± 0.34 ^a^	7.35 ± 1.25 ^a^	7.9 ± 1.05 ^a^	7.9 ± 1.08 ^a^	7.45 ± 0.98 ^a^	7.6 ± 0.97 ^a^
Texture	6.2 ± 1.03 ^c^	7.1 ± 1.1 ^b^	8.15 ± 0.63 ^a^	7.7 ± 0.98 ^ab^	8.1 ± 1.1 ^a^	8.35 ± 0.47 ^a^	8.2 ± 0.48 ^ab^	8.15 ± 0.34 ^a^
Aroma	8.9 ± 0.32 ^a^	8.9 ± 0.32 ^a^	8.9 ± 0.32 ^a^	8.9 ± 0.21 ^a^	8.9 ± 0.21 ^a^	8.9 ± 0.32 ^a^	8.65 ± 0.67 ^a^	8.75 ± 0.43 ^a^
Taste	7.3 ± 0.95 ^b^	7.3 ± 0.82 ^b^	8.2 ± 0.35 ^a^	8.1 ± 0.57 ^a^	8.1 ± 0.74 ^a^	8.2 ± 0.79 ^a^	8.2 ± 0.48 ^a^	7.95 ± 0.55 ^ab^
Overall acceptability	7.2 ± 0.79 ^b^	7.2 ± 0.92 ^b^	8.55 ± 0.50 ^a^	8.45 ± 0.50 ^a^	8.4 ± 0.52 ^a^	8.4 ± 0.52 ^a^	8.1 ± 0.74 ^a^	8.3 ± 0.48 ^a^

* Mean of three replicates ± standard deviation. Means that are followed by the same letter in the row and the same capital letter in the column did not differ significantly (*p* ˂ 0.05). **C2**: Control fermented milk by using commercial starter culture; **T4**: fermented milk by using AY8 as adjunct culture; **T5**: fermented milk by using AY15 as adjunct culture; **T6**: fermented milk by using mix of strains AY8 & AY15 as adjunct culture; **C1**: Control fermented milk by using commercial starter culture supplemented 2% JP; **T1**: fermented milk by using AY8 as adjunct culture supplemented 2% JP; **T2**: fermented milk by using AY15 as adjunct culture supplemented 2% JP; **T3**: fermented milk by using mix of strains AY8 & AY15 as adjunct culture supplemented 2% JP.

**Table 5:** Total phenol, flavonoid content, antioxidant activity, and acetyl choline esterase inhibition of functional jujuba fermented milk.

Properties	Samples (Mean ± SD *)							
	C2	T4	T5	T6	C1	T1	T2	T3
**Total** **phenols**								
Total phenols (mg gallic acid/100 g)	117.74 ± 0 ^d^	128.92 ± 2.53 ^c^	110.49 ± 1.66 ^e^	96.64 ± 0.52 ^f^	233.11 ± 0.92 ^a^	235.75 ± 1.92 ^a^	230.92 ± 3.23 ^a^	225.55 ± 1.53 ^b^
**Total Flavonoids**								
Total Flavonoids (mg RE/100 g)	1.21 ± 0.41 ^d^	14.07 ± 1.49 ^c^	11.93 ± 3.93 ^c^	6.93 ± 1.80 ^cd^	112.41 ± 4.54 ^a^	114.07 ± 4.54 ^a^	112.64 ± 3.93 ^a^	55.024 ± 7.15 ^b^
**Antioxidant activity**								
DPPH Scavenging activity (mg Ascorbic equivalent/100 g)	27.78 ± 0.71 ^d^	28.44 ± 0.55 ^d^	27.78 ± 2.68 ^d^	24.57 ± 3.76 ^d^	91.35 ± 7.76 ^b^	110.24 ± 6.11 ^a^	98.14 ± 1.03 ^b^	79.09 ± 2.73 ^c^
FRAP assay OH’-scavenged%	97.52 ± 0.05 ^c^	97.56 ± 0.05 ^c^	97.51 ± 0.05 ^c^	97.59 ± 0.05 ^c^	98.88 ± 0.05 ^b^	99.07 ± 0.05 ^a^	99.06 ± 0.05 ^a^	99.05 ± 0.05 ^a^
**Acetyl choline esterase inhibitors**								
Inhibition of Acetylcholine Esterase activity (%)	12.30 ± 0.55 ^g^	24.49 ± 0.44 ^c^	22.45 ± 0.77 ^d^	17.78 ± 1.02 ^f^	18.27 ± 0.17 ^f^	30.66 ± 0.72 ^a^	26.38 ± 0.25 ^b^	20.70 ± 0.89 ^e^

* Mean of three replicates ± standard deviation. Means that are followed by the same letter in the row and the same capital letter in the column did not differ significantly (*p* ˂ 0.05). **C2**: Control fermented milk by using commercial starter culture; **T4**: fermented milk by using AY8 as adjunct culture; **T5**: fermented milk by using AY15 as adjunct culture; **T6**: fermented milk by using mix of strains AY8 & AY15 as adjunct culture; **C1**: Control fermented milk by using commercial starter culture supplemented 2% JP; **T1**: fermented milk by using AY8 as adjunct culture supplemented 2% JP; **T2**: fermented milk by using AY15 as adjunct culture supplemented 2% JP; **T3**: fermented milk by using mix of strains AY8 & AY15 as adjunct culture supplemented 2% JP.

## Data Availability

The whole genome sequences of AY8 and AY15 were deposited at the DDBJ/ENA/GenBank database under the bioproject numbers PRJNA1330596 and PRJNA1330610, respectively. The data supporting this article have been included in the Supplementary File.

## Supplementary Materials

The following supporting information can be downloaded at: https://www.mdpi.com/2304-8158/14/24/4264/s1, Table S1: The source of traditional dairy products. Table S2: Screening of traditional lactobacilli isolates for inhibition of acetyl choline activity and their hemolytic activity. Table S3: Screening of traditional cocci isolated for inhibition of acetyl choline activity and their hemolytic activity. Table S4: Susceptibility of LAB isolates against some antibiotics and hemolysis activities of different strains.
