# Supplementary material for: Development and Genomic Evaluation of a Novel Functional Fermented Milk Formulated with Lactobacillus delbrueckii Strains and Jujuba Kernel Powder for Potential Neuroprotective Effects
Source: Foods. 2025 Dec 11;14(24):4264. doi: 10.3390/foods14244264 (PMC12754561; doi:10.3390/foods14244264)
Supplement: Supplementary file 1 [file foods-14-04264-s001.zip › foods-4011325-supplementary.pdf]

Table S1. The source of traditional dairy products

Type of dairy product	Source	Total Number of samples
Karish cheese	Egypt, Alexandria	7
	Egypt, El Behira	
	Egypt, El Gharbia	
Mish cheese	Egypt, El Behira,	7
	Egypt, Upper Egypt	
Laban Rayeb	Egypt, Elbehira	1
Ras Cheese	Egypt, Alexandria	4
Yalk milk	China, Caibuhe	6
	China,Buluobu	
	China,Kayangbao	
	China, Douhegengzhidi	
	China, Gangqihe	
	China, Mediduo	

Table S2. Screening of traditional lactobacilli isolates for inhibition of acetyl choline activity and their hemolytic activity

Isolate code	Product	Catalase	Gram stain	Mean±SD Acetyl choline esterase inhibition %	Heamolysis
AY1	Yak milk	-	+	30.26±2.77	-
AY2		-	+	45.85±1.24	γ
AY3		-	+	43.99 ± 2.10	γ
AY4		-	+	41.12±2.53	γ
AY5		-	+	41.79± 2.36	γ
AY8		-	+	46.99± 0.26	γ
AY10		-	+	31.34±1.45	-
AY11		-	+	45.55±1.86	γ
AY14		-	+	30.75±1.94	-
AY15		-	+	39.86±0.88	γ
AY16		-	+	37.08±1.82	γ
AY19		-	+	46.50 ± 1.46	γ
AY22		-	+	27.58±1.20	γ
ELA2	Laban Rayeb	-	+	32.39±1.89	γ
ELA3		-	+	36.04±1.89	γ
ELA5		-	+	46.37±0.51	B
EKA6	Karish cheese	-	+	13.05±1.16	-
EKA10		-	+	14.01±2.35	-
EKA16		-	+	14.98±2.84	-
EKA19		-	+	23.34±2.15	-
EKA20		-	+	16±2.09	-
EKA23		-	+	28.15±3.84	-
EKA26		-	+	26.97±2.50	-
EKA27		-	+	26.83±1.51	-
EKA28		-	+	44.21±1.24	B
EMA 2	Mish cheese	-	+	29.76±4.18	-
EMA3		-	+	26.15±1.71	-
EMA5		-	+	27.92±8.80	-
EMA6		-	+	22.68±1.79	-
EMA7		-	+	25.99±1.72	-
EMA8		-	+	45.42±0.31	B
EMA9		-	+	44.35±1.95	γ
EMA12		-	+	26.73±2.89	-
EMA13		-	+	24.42±0.38	-
EMA15		-	+	30.86±0.94	-
EMA16		-	+	38.27± 3.88	γ
EMA17	Mish cheese	-	+	31.23±1.36	-
EMA20		-	+	19.15±0.38	-
EMA22		-	+	31.44±4.54	-
EMA23		-	+	30.98±2.92	-
EMA24		-	+	45.46±1.57	B
ERA2	Ras cheese	-	+	29.11±1.16	-
ERA3		-	+	39.08±1.09	γ
ERA4		-	+	32.12±3.5	-
ERA5		-	+	30.68±2.3	-

Isolate code	product	Catalase	Gram stain	Mean±SD<br><br>Acetyl choline esterase inhibition %	Heamolysis
ERA7	Ras cheese	-	+	44.66±1.42	γ
ERA12		-	+	26.53±1.81	-
ERA13		-	+	34.77±3.17	-

Table S3. Screening of traditional cocci isolated for inhibition of acetyl choline activity and their hemolytic activity

Isolate code	Source	Catalase	Gram stain	Mean Acetyl choline esterase inhibition %	Heamolysis
AY6	Yalk milk	-	+	8.46±5.27	-
AY7		-	+	16.06±8.71	-
AY9		-	+	11.12±3.35	-
AY12		-	+	15.12±3.03	-
AY13		-	+	4.87±3.17	-
AY17		-	+	18.22±1.30	-
AY18		-	+	16.82±3.16	-
AY20		-	+	13.09±7.92	-
AY21		-	+	26.32±6.41	γ
ELA1	Laban Rayeb	-	+	20.49±4.62	γ
EKA1	Karish cheese	-	+	0±0	-
EKA3		-	+	8.46±5.27	-
EKA4		-	+	18.17±3.27	-
EKA7		-	+	0±0	-
EKA8		-	+	16.06±8.71	-
EKA9		-	+	3.33±2.54	-
EKA11		-	+	46.19±2.11	γ
EKA12		-	+	0±0	-
EKA13		-	+	0±0	-
EKA14		-	+	0±0	-
EKA15		-	+	7.43±2.76	-
EKA17		-	+	6.95±2.88	-
EKA18		-	+	18.28±3.30	-
EKA21		-	+	1.05±1.63	-
EKA22		-	+	5.17±1.78	-
EKA24		-	+	2.18±1.98	-
EKA25		-	+	8.94±2.69	-
EMA1	Mish cheese	-	+	10.25±2.40	-
EMA4		-	+	7.72±2.31	-
EMA10		-	+	7.85±2.31	-
EMA11		-	+	20.05±0.52	Γ
EMA14		-	+	6.61±2.84	-
EMA18		-	+	12.87±2.40	-
EMA19		-	+	8.46±0.14	-
EMA21		-	+	17.04±1.57	-
ERA1	Ras cheese	-	+	1.77±5.55	-
ERA6		-	+	15.1±4.83	-
ERA8		-	+	4.89±0.64	-
ERA9		-	+	0.38±4.25	-
ERA10		-	+	5.83±2.08	-
ERA11		-	+	0±0	-
ERA14		-	+	21.50±0.82	γ
ERA15		-	+	16.28±3.41	-
ERA16				4.87±8.14	-
ERA17				2.18±3.53	-
ERA18				17.04±1.57	-

Table S4. Susceptibility of LAB isolates against some antibiotics and hemolysis activities of different strains

Strain Code	Antibiotic susceptibility (inhibition zone in mm)									
	AMP	CTR	TET	GEN	PEN	E	C	CIP	MY	Sxt	N of antibiotic sensitive
AY2	S(37)	(S) 32	S (26)	R(11)	S(36)	S(28)	S(24)	R(0)	S25	R(0)	7/10
AY3	S(22)	(I)22	S(24)	R(10)	S(30)	S(28)	S(27)	R(0)	S27	R(0)	7/10
AY4	S(20)	(I)23	S(25)	R(10)	S(32)	S(28)	S(25)	R(0)	S25	R(0)	7/10
AY5	(S)23	(I)23	S(25)	R(10)	S(34)	S(28)	S(28)	R(0)	S25	R(0)	7/10
AY8	(S)31	S(30)	R(11)	S(26)	S(31)	S(25)	S(25)	R(11)	S26	I(13)	8/10
AY11	(S)32	S (26)	S(27)	R(12)	S(31)	I(15)	S(27)	R(9)	S25	I(12)	8/10
AY15	(S)35	(I)15	S(23)	R(11)	S(29)	S(29)	S(27)	R(0)	S25	R(0)	7/10
AY16	(S)33	(I)16	S(23)	R(11)	R(28)	S(28)	S(26)	R(0)	S23	R(0)	6/10
AY19	(S)22	(I)14	S(22)	S(17)	S(31)	S(25)	S(18)	R(0)	S17	R(10)	8/10
ELA3	(S)22	R (9)	S(16)	R(7)	R(18)	I(17)	R(12)	R(0)	S16	R(9)	4/10
EKA11	(S)30	(I)17	S(22)	S(16)	S(32)	S(25)	S(21)	R(6)	S24	S(17)	9/10
ERA3	(I)14	I (15)	R(9)	R(8)	S(32)	I(17)	S(19)	R(0)	R(0)	S(20)	6/10
ERA7	(S)31	(I)21	S(24)	S(17)	S(32)	I(20)	S(28)	R(12)	S28	S(24)	9/10
EMA9	(S)18	(I)17	R (0)	R(0)	R(20)	R(0)	S(21)	R(0)	R(0)	R(0)	3/10
EMA16	(S)23	(I)18	I (13)	R(11)	R(12)	I(17)	I(15)	R(0)	R(0)	S(27)	6/10

Antibiotic Susceptibility expressed as R (resistant), I (intermediate susceptibility), or S (susceptible). AMP, Ampicillin (10 µg); CTR, Ceflriaxone (30µg); TET, Tetracycline(30µg); GEN, Gentamicin (10µg); P, penicillin(10 U); E, erythromycin(15µg); CIP, Ciprofloxacin (5 µg); C, chloramphenicol(30 µg); MY, Lincomycin(2 µg)and Sxt, compound sulfamethoxa (25 µg)
